# Болезнь Иценко-Кушинга у ребенка с нетипичным дебютом болезни. Клинический случай с кратким обзором литературы

**DOI:** 10.14341/probl13102

**Published:** 2022-05-14

**Authors:** М. А. Тюльпаков, О. Б. Безлепкина, Е. В. Нагаева, В. Н. Азизян, А. М. Лапшина

**Affiliations:** Национальный медицинский исследовательский центр эндокринологии; Национальный медицинский исследовательский центр эндокринологии; Национальный медицинский исследовательский центр эндокринологии; Национальный медицинский исследовательский центр эндокринологии; Национальный медицинский исследовательский центр эндокринологии

**Keywords:** гиперкортицизм, болезнь Иценко–Кушинга, малая дексаметазоновая проба, большая дексаметазоновая проба, селективный забор крови из нижних каменистых синусов

## Abstract

Болезнь Иценко-Кушинга — это редкое мультисистемное заболевание, характеризующееся наличием эндогенного центрального гиперкортицизма вследствие АКТГ-секретирующей опухоли головного мозга. Частота болезни ­Иценко-Кушинга во взрослом возрасте составляет 0,7–2,4 на 1 млн населения, и лишь 10% всех случаев приходится на детский возраст. Возраст дебюта заболевания у детей составляет в среднем на 12,0–14,8 лет. Типичным проявлением заболевания у детей, наряду с ожирением и артериальной гипертензией, является снижение темпов роста. Золотым стандартом диагностики центрального гиперкортицизма служит МРТ головного мозга, однако эффективность данного метода у детей составляет лишь 50%. Основным методом лечения является нейрохирургическое трансназальное транссфеноидальное удаление эндоселлярной аденомы гипофиза, позволяющее достичь ремиссии более чем в 65% случаев. В данной статье описан клинический случай болезни Иценко-Кушинга у ребенка 6,5 лет с ожирением, артериальной гипертензией, нетипично «высоким» ростом, средними темпами роста и невизуализируемой кортикотропиномой. В статье представлены этапы диагностического поиска, сложности дифференциальной диагностики и хирургического лечения, результаты динамического наблюдения после проведенного лечения, а также представлен краткий обзор литературы.

## Abstract (en)

Itsenko–Cushing’s disease is a rare, multisystem disease characterized by the presence of endogenous central hypercortisolism due to an ACTH-secreting brain tumor. The frequency of Itsenko-Cushing’s disease in adulthood is 0.7–2.4 per 1 million population, and only 10% of all cases occur in childhood. The age of onset of the disease in children is on average 12.0–14.8 years. A typical manifestation of the disease in children, along with obesity and arterial hypertension, is a decrease in growth rates. The gold standard for diagnosing central hypercortisolism is MRI of the brain, however, the effectiveness of this method in children is only 50%. The main method of treatment is neurosurgical transnasal transsphenoidal removal of endosellar pituitary adenoma, which makes it possible to achieve remission in more than 65% of cases. This article describes a clinical case of Itsenko–Cushing’s disease in a 6.5-year-old child with obesity, arterial hypertension, atypically «high» stature, average velocity and non-visualizable corticotropinoma. The article presents the stages of diagnostic search, the complexity of differential diagnosis and surgical treatment, the results of follow-up after the treatment and a brief review of the literature.

## ВВЕДЕНИЕ

Синдром Иценко–Кушинга — редкое эндокринное заболевание у детей (2–5 случаев на 1 млн детского населения). Частота возникновения новых случаев заболевания составляет 0,7–2,4 на 1 млн населения, лишь 10% из них приходится на болезнь Иценко–Кушинга с дебютом в детском возрасте — заболевание, причиной которого является АКТГ-секретирующая аденома гипофиза (кортикотропинома), обуславливающая гиперсекрецию кортизола пучковой зоной коры надпочечников. Частота встречаемости у детей составляет 0,12–0,24 случая на 1 млн [1–4].

Как в педиатрической, так и в терапевтической практике кортикотропинома является самой частой причиной эндогенного гиперкортицизма (75–80% случаев), дебют заболевания, как правило, приходится на возраст старше 6 лет [5][6]. Среди гормонально-активных аденом гипофиза кортикотропиномы составляют 4–8% [7]. Пациенты с гиперкортицизмом обычно имеют характерный внешний вид и многочисленные клинические проявления, несмотря на это, диагноз часто бывает установлен лишь спустя месяцы или даже годы после первого обращения к врачу: от появления первых признаков болезни до постановки диагноза в среднем проходит 2,5±1,7 года [8]. Наиболее частыми жалобами у детей с гиперкортицизмом являются задержка роста и прогрессирующий набор массы тела.

Приводим клинический случай — описание ребенка с отсутствием «классических» признаков гипер­кортицизма, нормальными показателями роста и не визуализируемой кортикотропиномой гипофиза на момент диагностики заболевания.

Пациент М., 6,5 лет, впервые обследован в ФГБУ «НМИЦ эндокринологии» Минздрава России (далее — Центр) в возрасте 6 лет 6 месяцев с жалобами на избыточную массу тела, эпизоды повышения артериального давления (АД).

Анамнез жизни: мальчик от 2-ой нормально протекавшей беременности, срочных родов. Ранний постнатальный период — без особенностей. Ребенок от высоких родителей (целевой рост 192,5±10,0 см, SDS целевого роста +2,2), наследственность по материнской линии отягощена по сахарному диабету 2-ого типа — у бабушки.

Анамнез заболевания: с 4,5 лет у ребенка отмечалась избыточная прибавка массы тела, по месту жительства диагностировано экзогенно-конституциональное ожирение 2-ой степени, даны рекомендации по питанию. В течение последующих полутора лет у мальчика наблюдался прогрессирующий набор массы тела (около 1–2 кг в месяц), за 17 месяцев прибавка массы тела в общей сложности превысила 20 кг.

Ребенок впервые стационарно обследован по месту жительства в возрасте 6 лет 4 месяцев: рост 121,5 см, SDS роста +1,09; масса тела 49 кг, SDS индекса массы тела (ИМТ) +5,12; гликированный гемоглобин 6,0%. «Костный возраст» опережал хронологический на 6 месяцев. По данным суточного мониторирования артериального давления (СМАД) отмечались повышения АД до 145/80 мм рт.ст. При проведении МРТ головного мозга заподозрена микроаденома гипофиза, в связи с чем было рекомендовано обследование в ФГБУ «НМИЦ эндокринологии».

Впервые обследован в ФГБУ «НМИЦ эндокринологии» в возрасте 6 лет 6 месяцев. При поступлении состояние ребенка было относительно удовлетворительное, рост выше среднего (рост 123,8 см, SDS роста +1,10), отмечалось выраженное ожирение (масса тела 53,3 кг, SDS ИМТ +4,93) с равномерным распределением подкожножировой клетчатки, наличием черного акантоза в области подмышечных впадин, локтевых суставов и складок шеи, отсутствием стрий. АД 140/80 мм рт.ст., пульс 100 в минуту. В связи с выраженной прибавкой массы тела, наличием микроаденомы гипофиза в анамнезе была заподозрена болезнь Иценко–Кушинга.

Базальные уровни гормонов представлены в таблице 1.

**Table:** Таблица 1. Базальные уровни гормонов у пациента в возрасте 6,5 летTable 1. Basal hormone levels in a patient aged 6.5 years Примечание. АКТГ — адренокортикотропный гормон; ТТГ — тиреотропный гормон; св.Т4 — свободный тироксин; ИФР-1 — инсулиноподобный фактор роста 1 типа; ДГЭА-С — дегидроэпиандростерон-сульфат.

Показатель	Результат	Референсные интервалы
ТТГ, мМЕ/мл	0,987	0,51–4,82
св.Т4, пмоль/л	12,53	11,2–18,6
ИФР-1, нг/мл	311,8	17–347
ДГЭА-С, мкмоль/л	2,69	0,08–2,31
Пролактин, мЕд/л	309,5	78–380
Инсулин, мкЕ/мл	38,58	2,6–24,9
АКТГ, пг/мл	утро	вечер	утро	вечер
73,04 47,6	122,5 71,5	7,2–63,3	2,0–25,5
Кортизол в крови, нмоль/л	673,4 645,2	740,2 223,3	77,0–630,0	64,0–327,0
Кортизол в слюне, нмоль/л	14,56	0,5–9,65
Св. кортизол в суточной моче, нмоль/л	1093,5	100,0–379,0

Обращали на себя внимание умеренно повышенные утренние уровни кортизола и АКТГ (взятые в разные дни), однократно выявленное нарушение циркадного ритма секреции АКТГ и кортизола. В связи с повышенным уровнем инсулина ребенку был проведен оральный глюкозотолерантный тест, выявивший инсулинорезистентность (индекс HOMA 3,86, индекс Сaro 0,19) и нормогликемию.

Учитывая повышенные базальные уровни АКТГ и кортизола, нарушение циркадного ритма секреции этих гормонов, высокий уровень свободного кортизола в суточной моче, выраженное ожирение и наличие артериальной гипертензии, был заподозрен эндогенный гиперкортицизм, который предстояло подтвердить или опровергнуть.

При проведении малой дексаметазоновой пробы значимого подавления уровня кортизола и АКТГ ­получено не было, что свидетельствовало в пользу синдрома Иценко – Кушинга (табл. 2).

**Table:** Таблица 2. Результаты проведения функциональных проб у пациента в возрасте 6,5 летTable 2. Results of functional tests in a patient aged 6.5 years

Показатель	АКТГ, кровь, пг/мл	Кортизол, кровь, нмоль/л	Свободный кортизол в суточной моче, нмоль/л
Базальные уровни	73,0447,6	673,4645,2	1093,5
Малая дексаметазоновая проба	11,45	430,1	40,0
Большая дексаметазоновая проба	25,7	84,8	31,2

Следующим этапом поиска с целью дифференциальной диагностики между центральным и периферическим гиперкортицизмом было проведение большой дексаметазоновой пробы (табл. 2). Результаты теста свидетельствовали о центральном генезе заболевания: наблюдалось значимое снижение уровня свободного кортизола в суточной моче и кортизола в крови.

При повторном проведении МРТ головного мозга убедительных данных за наличие аденомы гипофиза получено не было, однако при контрастном усилении отмечалось локальное снижение контрастирования в правой части гипофиза. Учитывая отсутствие четко визуализируемого образования в хиазмально-селлярной области, с целью возможного наличия АКТГ-эктопированного образования была проведена мультиспиральная компьютерная томография грудной и брюшной полости: новообразования не выявлены.

Таким образом, у ребенка, имеющего нормальные темпы роста, в результате комплексного лабораторно-инструментального обследования источник гиперкортицизма обнаружен не был. Возникла гипотеза о наличии резистентности к глюкокортикостероидам, в связи с чем взят образец крови для поиска мутаций в гене NRC31, кодирующем белок глюкокортикоидного рецептора. На время ожидания результатов генетического исследования мальчику была назначена антигипертензивная терапия, и он был выписан под наблюдение педиатра и детского эндокринолога.

Повторная госпитализация осуществлена через 4 месяца, к этому времени был готов молекулярно-генетический анализ, однако патогенных, вероятно патогенных и вариантов неопределенной клинической значимости в гене NRC31 обнаружено не было. При осмотре обращало на себя внимание изменение внешности ребенка, произошедшее с момента предыдущей госпитализации (рис. 1), появление перераспределения жировой клетчатки (избыточное ее отложение в области щек и возникновение климактерического горбика) при крайне незначительной прибавке массы тела (200 г за 4 месяца), наличие акне на лице, а главное — появление снижения темпов роста: за 4 месяца вырос на 0,8 см (рост 124,6 см), SDS скорости роста -2,3.

**Figure:**
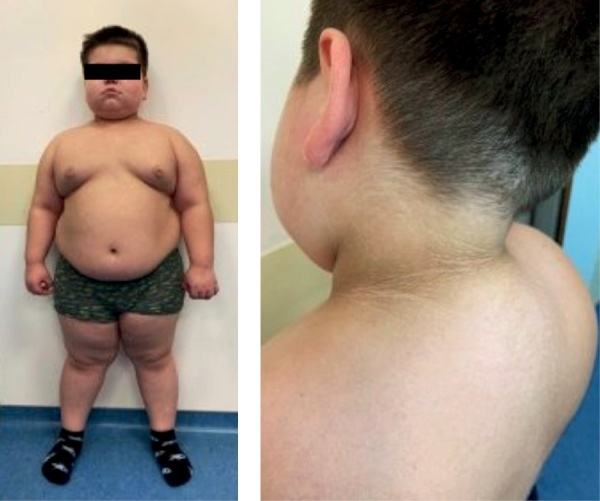
Рисунок 1. Фенотипические особенности пациента в 6,5 лет: перераспределение подкожножировой клетчатки, наличие климактерического горбика, гиперпигментация кожных складок.Figure 1. Phenotypic features of a patient at 6.5 years old: redistribution of subcutaneous fat, the presence of a climacteric hump, hyperpigmentation of skin folds.

Учитывая отсутствие мутации в гене NRC31, снижение темпов роста, появление перераспределения жировой ткани, было принято решение о повторном проведении детального обследования по поводу гиперкортицизма.

Обнаружение повышенных концентраций кортизола и АКТГ в крови, кортизола в слюне, свободного кортизола в суточной моче, а также нарушения циркадного ритма секреции этих гормонов подтвердили наличие у ребенка гиперкортицизма (табл. 3).

**Table:** Таблица 3. Базальные уровни гормонов при повторном обследовании в возрасте 6 лет 9 месяцевTable 3. Basal hormone levels at follow-up at age 6 years 9 months

Показатель	Результат	Референсные интервалы
АКТГ, пг/мл	утро	вечер	утро	вечер
51,1	63,3	7,2–63,3	2,0–25,5
Кортизол в крови, нмоль/л	412,4	479,4	77,0–630,0	64,0–327,0
Кортизол в слюне, нмоль/л),	10,8	0,5–9,65
Св. кортизол в суточной моче, нмоль/л	1093,5	100,0–379,0

С целью дифференциальной диагностики между функциональным и патологическим гиперкортицизмом повторно были проведены малая и большая дексаметазоновые пробы. По результатам малой дексаметазоновой пробы отсутствовало подавление уровней кортизола и АКТГ крови, свободного кортизола в суточной моче, что, безусловно, подтверждало наличие у ребенка патологического гиперкортицизма. Данные большой дексаметазоновой пробы подтвердили АКТГ-зависимый характер гиперкортицизма: отмечалось значимое ­подавление уровней кортизола и АКТГ в крови, свободного кортизола — в суточной моче (табл. 4).

**Table:** Таблица 4. Результаты повторного проведения функциональных проб у пациента в возрасте 6 лет 9 месяцевTable 4. Results of repeated functional tests in a patient aged 6 years 9 months

Показатель	АКТГ, кровь, пг/мл	Кортизол, кровь, нмоль/л	Свободный кортизол в суточной моче, нмоль/л
Базальные уровни	51,1	412,4	1117,5
Малая дексаметазоновая проба	48,1	268,1	47,7
Большая дексаметазоновая проба	12,9	21,7	47,6

МРТ головного мозга, как и в предыдущий раз, выявила локальное снижение контрастирования в правой части аденогипофиза, однако убедительных данных за наличие аденомы гипофиза вновь получено не было, по сравнению с исследованием от2020 г. существенной динамики не отмечалось (рис. 2).

**Figure:**
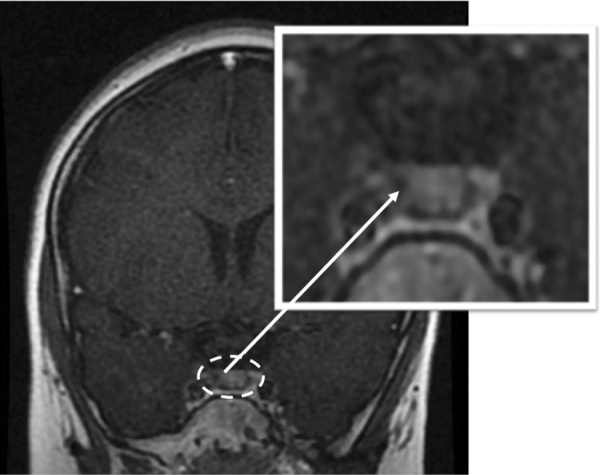
Рисунок 2. МРТ головного мозга в 6 лет 9 месяцев. Пунктирной линией выделен аденогипофиз, стрелкой указано локальное снижение контрастирования в правой части аденогипофиза, убедительных данных за наличие аденомы гипофиза нет.Figure 2. Brain MRI at  6 years 9 months. The dotted line indicates the adenohypophysis, the arrow indicates a local decrease in contrast in the right side of the adenohypophysis, there is no convincing evidence for the presence of pituitary adenoma.

Таким образом, учитывая значимое снижение темпов роста (SDS роста -2,3), появление фенотипических признаков, характерных для гиперкортицизма (перераспределение жировой клетчатки, появление акне), нарушение циркадного ритма секреции кортизола и АКТГ, несмотря на отсутствие явных признаков аденомы гипофиза, диагноз АКТГ-зависимый эндогенный гиперкортицизм не вызывал сомнений. Принято решение о проведении трансназального, транссфеноидального удаления аденомы гипофиза с применением эндоскопического ассистирования справа.

К сожалению, оперативное вмешательство не увенчалось успехом: в послеоперационном периоде сохранялся гиперкортицизм (в 1-е сутки после операции: АКТГ — 87,57 пг/мл, кортизол — 1888 нмоль/л, 2-е: АКТГ — 113,1 пг/мл, кортизол — 1179 ммоль/л). Данные гистологического исследования послеоперационного материала гипофиза не подтвердили наличие аденомы, на дополнительных срезах с импрегнацией серебром обнаружены фрагменты аденогипофиза с сохранной сетью ретикулиновых волокон.

Учитывая неэффективность проведенного нейрохирургического вмешательства, с целью уточнения локализации кортикотропиномы было принято решение о проведении селективного забора образцов крови из нижних каменистых синусов для исследования в них уровней АКТГ. Последовательно были выполнены селективные заборы крови из нижних каменистых синусов до и после стимуляции десмопрессином в дозе 4 мкг (через 3, 5 и 10 мин после стимуляции), результаты представлены в таблице 5.

**Table:** Таблица 5. Результаты селективного забора крови из нижних каменистых синусов на фоне стимуляции десмопрессиномTable 5. Results of selective blood sampling from the inferior petrosal sinuses against the background of stimulation with desmopressin

Уровень АКТГ
Время	Правыйсинус	Периферическая вена	Левый синус	Максимальный градиент между центром и периферической веной до и после стимуляции	Максимальный градиент между правым и левым синусами до и после стимуляции
-5 мин	191,7	125,4	1137	9,06	5,93
0 мин	139	116,4	725,6	6,23	5,2
+3 мин	154,4	130,2	1475	11,3	9,55
+5 мин	184.4	139,5	1813	12,9	9,83
+10 мин	206,9	154,5	1047	6,78	5,06
Уровень пролактина
-5 мин	687,5	164,9	1454	8,81	2,11

Выявлен значительный (более 3) градиент концентрации АКТГ в левом синусе и периферической вене. Учитывая результаты селективного забора крови, однозначно подтверждающие наличие гиперпродукции АКТГ левой доли аденогипофиза, через 7 дней после первой операции было проведено повторное трансназальное, транссфеноидальное удаление эндоселлярной аденомы гипофиза с применением эндоскопического ассистирования.

Гистологическое исследование послеоперационного материала подтвердило наличие аденомы в удаленных тканях (рис. 3).

**Figure:**
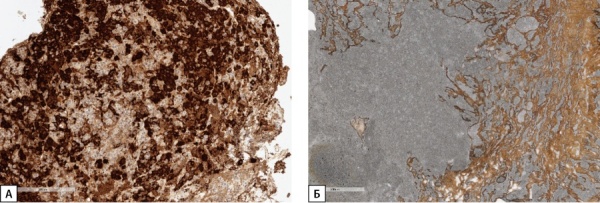
Рисунок 3. Гистологическое исследование удаленной аденомы гипофиза.Figure 3. Histological examination of a resected pituitary adenoma. А — выраженное окрашивание цитоплазмы всех опухолевых клеток с антителами к АКТГ и СAM 5.2 (моноклональные антитела к цитокератинам САМ 5.2). Экспрессия Ki-67 не обнаружена. Б — разрушенная сеть ретикулиновых волокон в опухоли и сохранная сеть в ткани передней доли гипофиза (импрегнация серебром).

При гистохимическом исследовании срезов (импрегнация серебром) выявлена разрушенная сеть ретикулиновых волокон в опухоли и сохранная сеть — в ткани передней доли гипофиза. При иммуногистохимическом исследовании обнаружено выраженное окрашивание цитоплазмы практически всех опухолевых клеток с антителами к АКТГ и САМ 5.2. Экспрессия Ki-67 не обнаружена.

На следующие сутки после операции у мальчика наблюдалось снижение уровня АКТГ до 8,94 пг/мл, кортизола — до 13.52 нмоль/л. Таким образом, в результате повторного нейрохирургического вмешательства цель была достигнута: удален источник гиперкортицизма, кроме того, развился вторичный гипотиреоз: концентрация св.Т4 — 9,5 пмоль/л. Ребенок был выписан домой на заместительной терапии препаратами гидрокортизона и левотироксина натрия, по результатам СМАД необходимости в продолжении антигипертензивной терапии не было.

При контрольной госпитализации через 6 мес в возрасте 7 лет 3 месяцев у ребенка наблюдалась нормализация темпов роста: рост 129,9 (SDS роста +1,3, SDS скорости роста +4,37) (рис. 4).

**Figure:**
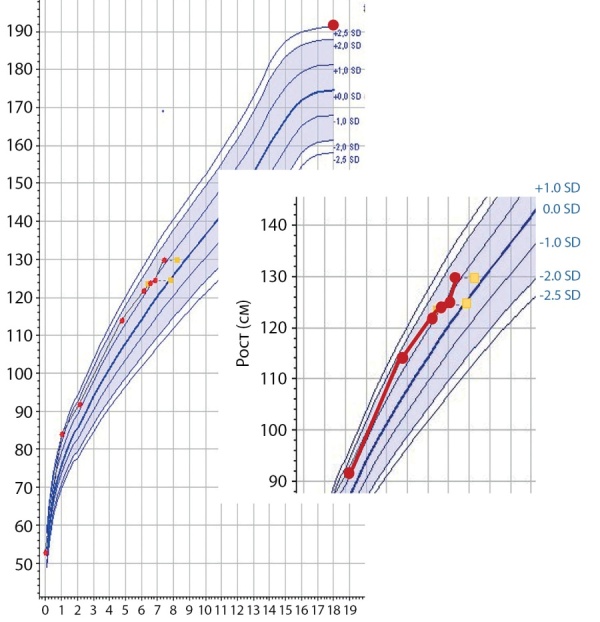
Рисунок 4. Кривая роста через 6 месяцев после хирургического лечения: восстановление темпов роста. Желтые квадратики — костный возраст.Figure 4. Growth curve 6 months after surgery: restoration of growth rates. Yellow squares — bone age.

Обращали на себя внимание выраженное снижение массы тела (минус 5,3 кг за 6 мес, ∆ SDS ИМТ -0,83), равномерное распределение подкожножировой клетчатки, исчезновение климактерического горбика, акне на лице, а также выраженное снижение интенсивности акантоза (рис. 5).

**Figure:**
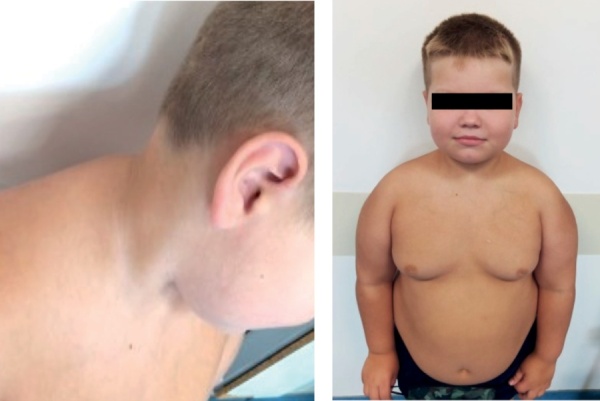
Рисунок 5. Пациент при динамическом обследовании: отсутствие перераспределения жировой клетчатки, отсутствие акне и климактерического горбика, выраженное снижение интенсивности акантоза.Figure 5. Patient during dynamic examination: no redistribution of adipose tissue, no acne and menopausal hump, a pronounced decrease in the intensity of acanthosis.

Утренние уровни АКТГ и кортизола составили 15,35 пг/мл и 5,64 нмоль/л соответственно. Мальчик был декомпенсирован по вторичному гипотиреозу (св.Т4 — 10,71 пмоль/л (11,2–18,6)), в связи с чем терапия левотироксином натрия была скорректирована. Данных за нарушение углеводного обмена получено не было (ISI Matsuda — 5,25; Сarо — 0,33; HOMA — 2,18). У ребенка отмечались высокие цифры АД (максимальное САД — 141 мм рт. ст.), по всей видимости, связанные с избыточным весом, однако выявленная лабильная артериальная гипертензия не требовала постоянной антигипертензивной терапии, кардиологом даны рекомендации по контролю АД и дальнейшему снижению веса.

Таким образом, можно говорить об успешном лечении пациента, однако не исключен риск возможных отдаленных осложнений, таких как дефицит соматотропного гормона, гипогонадотропный гипогонадизм, в связи с чем ребенок нуждается в длительном динамическом наблюдении детским эндокринологом.

## ОБСУЖДЕНИЕ

Болезнь Иценко–Кушинга является наиболее частой причиной эндогенного гиперкортицизма у детей в возрасте старше 7 лет, доля которого составляет 75–80%. Как правило, болезнь Иценко–Кушинга возникает в предподростковом или подростковом возрасте, средний возраст дебюта заболевания находится в возрастном диапазоне 12,0–14,8 лет [9]. Для детей младше 7 лет наиболее частыми причинами гиперкортицизма являются заболевания надпочечников (аденома, адренокортикальный рак, двусторонняя гиперплазия надпочечников, макронодулярная гиперплазия надпочечников, синдром Мак-Кьюна–Олбрайта–Брайцева).

Жалобы у пациентов с болезнью Иценко–Кушинга весьма разнообразны и могут затрагивать практически все органы и системы. При синдроме Кушинга отмечается перераспределение жировой ткани по абдоминальному типу, характерно ее отложение на животе и груди, в височных ямках и щеках («лунообразное лицо»), формирование жировых подушек над ключицами и в области VII шейного позвонка — образование «климактерического горбика» [7][8].

Задержка роста — один из наиболее значимых и ранних признаков синдрома Иценко–Кушинга в детском возрасте, в большинстве случаев она предшествует появлению других симптомов заболевания. Избыток глюкокортикоидов подавляет рост посредством нескольких механизмов:

Чем меньше возраст заболевшего ребенка и больше продолжительность заболевания до момента диагностики, тем больше выражена задержка роста. По данным Н.А. Стребковой и соавт., у детей с болезнью Иценко–Кушинга на момент диагностики заболевания SDS роста составлял от -0,7 до -4,2 (при костном возрасте не более 13 лет).

Нарушение роста традиционно считается ключевым клиническим признаком гиперкортицизма у детей. В серии исследований у 95% пациентов SDS роста был ниже 0 SD, однако только 37% из 52 детей с синдромом Кушинга на момент постановки диагноза имели выраженную (SDS роста ≤2) задержку роста [4].

Большинство образований хиазмально-селлярной области у детей — микроаденомы диаметром до 5 мм. АКТГ­секретирующие макроаденомы в детском возрасте встречаются реже, чем у взрослых (2 и 15% случаев соответственно) [10].

Диагностика болезни Иценко–Кушинга базируется на характерной клинической картине заболевания, данных гормонального обследования и результатах МРТ головного мозга. Прежде всего необходимо доказать наличие гиперкортицизолизма. Ни один из существующих тестов не обладает 100% точностью, у каждого есть свои ограничения, и в большинстве случаев необходимо проведение нескольких исследований для постановки диагноза [10][11].

Наиболее удобным методом диагностики эндогенного гиперкортицизма является определение кортизола в слюне, собранной в 2300. Сбор слюны может выполняться самостоятельно пациентом в амбулаторных условиях, методика минимизирует вероятность стресса. Слюна содержит свободный кортизол, уровень которого не зависит от содержания кортизолсвязывающего глобулина и количества слюны, что дает преимущество перед определением свободного кортизола в суточной моче, когда потеря части мочи влияет на результат [12][13].

Крайне информативным является определение суточного ритма кортизола и АКТГ крови. В норме суточный ритм секреции кортизола определяется гормональной активностью кортикотрофов гипофиза. Их секреторная активность начинает увеличиваться во второй половине ночи, достигает максимума в ранние утренние часы, затем постепенно снижается в течение первой половины дня. В норме вечерний уровень кортизола должен быть ниже утренних показателей на 50% и более. В нашем клиническом случае, учитывая данные анамнеза, объективного осмотра и заподозренный диагноз, с целью минимизации инвазивных исследований (забор крови) у ребенка было принято решение об однодневном сборе суточного ритма кортизолаи АКТГ, а также ночной слюны на кортизол. К сожалению, в связи с технической ошибкой с первого раза не удалось собрать мочу на суточную экскрецию кортизола, поэтому повторные базальные исследования были выполнены через 7 дней после завершения большой дексаметазоновой пробы.

Согласно клиническим рекомендациям по диагностике болезни Иценко–Кушинга, «оправдано проведение как минимум 2-х тестов 1-й линии (определение кортизола в слюне, собранной в 23:00; кортизола в сыворотке крови, взятой утром после приема 1 мг дексаметазона, произведенного накануне в 23:00). При дискордантном результате 2-х первых тестов показано проведение дополнительных исследований (свободный кортизол в суточной моче, определение кортизола в крови вечером)» [12]. Однако, поскольку ни один из этих тестов не обладает 100% диагностической точностью, в педиатрической практике принято использовать несколько диагностических тестов с целью подтверждения диагноза [13].

Дексаметазон является высокоактивным глюкокортикоидом, способным в небольшой концентрации подавлять секрецию АКТГ гипофизом. При подозрении на синдром Иценко–Кушинга для дифференциальной диагностики между функциональным ипатологическим гиперкортицизмом проводятся супрессивные тесты с дексаметазоном. Для данной цели обычно используется ночной тест с 1 мг дексаметазона (1 мг в 23:00), однако в педиатрической практике за счет отсутствия исследований набольших группах детей наравне с ночным тестом используется малая дексаметазоновая проба. Помимо этого, малая дексаметазоновая проба (пациент суммарно получает 4 мг дексаметазона: по 0,5 мг каждые 6 ч в течение 48 ч) обладает большей чувствительностью в сравнении с ночным тестом [13].

Следующим шагом в дифференциальной диагностике между болезнью Иценко–Кушинга и другими формами гиперкортицизолизма является большая дексаметазоновая проба (пациент получает суммарно 16 мг дексаметазона: по 2 мг каждые 6 ч в течение 48 ч) [12][13].

Золотым стандартом диагностики, особенно при подозрении на АКТГ-зависимый гиперкортицизм, является МРТ головного мозга [14]. Оптимальная толщина среза должна составлять 2–3 мм. Т2­взвешенные изображения позволяют визуализировать кистозные компоненты после введения контраста, однако эффективность данного метода у детей составляет лишь 50% [14].

Когда результат МРТ головного мозга не подтверждает наличие опухоли, рекомендовано проведение одномоментного двустороннего селективного забора крови из нижних каменистых синусов на фоне стимуляции аналогом кортиколиберина. Среди всех возможных методов дифференциальной диагностики АКТГ-зависимых форм гиперкортицизма селективный забор крови из нижних каменистых синусов считается наиболее точным. Он позволяет дифференцировать болезнь Иценко–Кушинга отАКТГ-эктопии. В начале 1990-х E.H. Oldfield и соавт. предложили использовать кортиколиберин для повышения чувствительности и специфичности метода [15][16]. Для определения положения катетера было предложено, наряду с АКТГ, исследовать содержание других тропных гормонов [17-20]. Учитывая, что концентрации ТТГ, соматотропного гормона могут подавляться у пациентов с болезнью Иценко–Кушинга, а также типичное расположение нормальных лактотрофов в гипофизе, редко вовлеченных в патологический рост аденомы, пролактин является основным маркером успешной катетеризации нижних каменистых синусов [18-22]. Учитывая импульсный характер секреции АКТГ, несколько образцов крови берутся одновременно из обоих синусов и из периферической вены, затем вводится кортиколиберин (1 мкг/кг массы тела), и несколько образцов крови забираются на фоне стимуляции. Многочисленные исследования показали, что градиент ≥2 между центром и ­периферией достимуляции надежно свидетельствует о болезни Иценко–Кушинга [15]. После стимуляции кортиколиберином градиент АКТГ центр/периферия ≥3 еще более точно подтверждает диагноз — болезнь Иценко–Кушинга. У большинства пациентов с синдромом АКТГ-эктопии градиент между центром и периферией выявить не удается или этот градиент меньше 2-х как исходно, так и после стимуляции [15][16][23][24].

Учитывая неагрессивное течение заболевания (что нехарактерно для АКТГ-эктопии), наличие зоны локально сниженного контрастирования по данным МРТ головного мозга, ранний возраст ребенка, риски при проведении селективного забора, у этого ребенка консилиумом врачей было вынесено решение, что «диагноз — болезнь Иценко–Кушинга не вызывает сомнений, показаний к проведению селективного забора нет». И мальчик был прооперирован. К сожалению, как было изложено выше, эффекта от оперативного лечения не было. При проведении повторного консилиума с учетом персистенции АКТГзависимого гиперкортицизма, отсутствия данных за аденому гипофиза по данным морфологического исследования, а также отсутствия четких данных за аденому гипофиза по предоперационному МРТ, было принято решение о проведении селективного забора крови из нижних каменистых синусов на фоне стимуляции десмопрессином для решения вопроса о тактике дальнейшего лечения.

Всем пациентам с установленным диагнозом «болезнь Иценко–Кушинга» должно быть проведено нейрохирургическое лечение (эндоскопическая трансназальная аденомэктомия), позволяющее достичь ремиссии в 65–90% случаев [10][25][26]. Вероятность ремиссии выше у пациентов с микроаденомой гипофиза и во многом зависит от опыта нейрохирурга и возможности полностью удалить ткань опухоли. Существует взаимосвязь между количеством операций в год и процентом ремиссии у пациентов с БИК в отдельных специализированных центрах [10][25][26]. В случае неэффективности первой операции или рецидива заболевания возможно проведение повторного нейрохирургического вмешательства. Иногда уровень кортизола снижается постепенно, что обусловлено возникшей автономией работы надпочечников [10][25][26].

Анализируя данный клинический случай, можно констатировать тот факт, что проведение селективного забора перед первой операцией не изменило бы подход или кратность хирургических вмешательств у ребенка, так как первоначальная нейрохирургическая тактика была бы в любом случае нацелена на удаление доли гипофиза, где отмечалось снижение локального контрастирования, а сам селективный забор не помог уточнить точной локализации аденомы.

## ЗАКЛЮЧЕНИЕ

Представленный клинический случай демонстрирует возможность появления болезни Иценко–Кушинга в столь юном возрасте, а также сложности ее диагностики, обусловленные нетипичным для данного заболевания ранним началом; нетипичной клинической картиной болезни Иценко–Кушинга: отсутствие задержки роста, нормальная скорость роста, стертые фенотипические проявления, наличие невизуализируемой кортикотропиномы. Данные особенности, вероятно, объясняются диагностикой заболевания в самом его дебюте.

## ДОПОЛНИТЕЛЬНАЯ ИНФОРМАЦИЯ

Источники финансирования. Работа выполнена по инициативе авторов без привлечения финансирования.

Конфликт интересов. Авторы декларируют отсутствие явных и потенциальных конфликтов интересов, связанных с содержанием настоящей статьи.

Участие авторов. Все авторы одобрили финальную версию статьи перед публикацией, выразили согласие нести ответственность за все аспекты работы, подразумевающую надлежащее изучение и решение вопросов, связанных с точностью или добросовестностью любой части работы.

Согласие пациента. Пациент добровольно подписал информированное согласие на публикацию персональной медицинской информации в обезличенной форме.

## References

[cit1] Acharya Runa, Kabadi Udaya M (2017). Case of diabetic ketoacidosis as an initial presentation of Cushing’s syndrome. Endocrinology, Diabetes & Metabolism Case Reports.

[cit2] Stratakis CA. Cushing Syndrome in Pediatrics. Endocrinol Metab Clin North Am. 2012;41(4):793-803. doi: https://doi.org/10.1016/j.ecl.2012.08.002 23099271 PMC3594781

[cit3] Yanar E. A., Makazan N. V., Orlova E. M., Kareva M. А. (2021). Genetic basis of Cushing’s disease in children and targeted therapeutic future perspectives. Problems of Endocrinology.

[cit4] Nayak S, Dabadghao P, Dixit P, et al. Cushing’s disease in children: a review. Neurol India. 2020;68(Supplement):S52-S65. doi: https://doi.org/10.4103/0028-3886.287677 32611893

[cit5] Stratakis Constantine A. (2018). An update on Cushing syndrome in pediatrics. Annales d'Endocrinologie.

[cit6] Storr Helen L, Savage Martin O (2015). MANAGEMENT OF ENDOCRINE DISEASE: Paediatric Cushing's disease. European Journal of Endocrinology.

[cit7] Pivonello R, De Leo M, Cozzolino A, Colao A. The treatment of Cushing’s disease. Endocr Rev. 2015;36(4):385-486. doi: https://doi.org/10.1210/er.2013-1048 26067718 PMC4523083

[cit8] Solntseva A.V. Giperkortitsizm u detei. Uchebno-metodicheskoe posobie. — Minsk: BGMU; 2020. C. 19.

[cit9] Storr Helen L., Chan Li F., Grossman Ashley B., Savage Martin O. (2007). Paediatric Cushing's syndrome: epidemiology, investigation and therapeutic advances. Trends in Endocrinology & Metabolism.

[cit10] Pappachan Joseph M, Hariman Christian, Edavalath Mahamood, Waldron Julian, Hanna Fahmy W (2017). Cushing's syndrome: a practical approach to diagnosis and differential diagnoses. Journal of Clinical Pathology.

[cit11] Dedov I.I., Peterkova V.A. Rukovodstvo po detskoi endokrinologii. — M.: UNIVERSUM PABLIShING; 2006. C. 117-131.

[cit12] Melnichenko G A, Dedov I I, Belaya Zh E, Rozhinskaya L Ya, Vagapova G R, Volkova N I, Grigor’ev A Yu, Grineva E N, Marova E I, Mkrtumayn A M, Trunin Yu Yu, Cherebillo V Yu (2015). Cushing’s disease: the clinical features, diagnostics, differential diagnostics, and methods of treatment. Problems of Endocrinology.

[cit13] Ferrigno Rosario, Hasenmajer Valeria, Caiulo Silvana, Minnetti Marianna, Mazzotta Paola, Storr Helen L, Isidori Andrea M, Grossman Ashley B, De Martino Maria Cristina, Savage Martin O (2021). Paediatric Cushing’s disease: Epidemiology, pathogenesis, clinical management and outcome. Reviews in Endocrine and Metabolic Disorders.

[cit14] Sitkin Ivan I., Malygina Anastasia A., Belaya Zhanna E., Rozhinskaya Lyudmila Y., Buryakina Svetlana A. (2018). Inferior petrosal sinus sampling in differential diagnosis of ACTH-dependent hypercortisolism. Endocrine Surgery.

[cit15] Juul Kristian Vinter, Bichet Daniel G., Nørgaard Jens Peter (2011). Desmopressin duration of antidiuretic action in patients with central diabetes insipidus. Endocrine.

[cit16] Juul Kristian Vinter, Erichsen Lars, Robertson Gary L. (2012). Temporal delays and individual variation in antidiuretic response to desmopressin. American Journal of Physiology-Renal Physiology.

[cit17] ALLOLIO BRUNO, GÜNTHER ROLF W., BENKER GEORG, REINWEIN DANKWART, WINKELMANN WERNER, SCHULTE HEINRICH M. (2009). A Multihormonal Response to Corticotropin-Releasing Hormone in Inferior Petrosal Sinus Blood of Patients with Cushing's Disease. The Journal of Clinical Endocrinology & Metabolism.

[cit18] McNally Paul G., Bolia Amman, Absalom Steve R., Falconer-Smith James, Howlett Trevor A. (2008). Preliminary observations using endocrine markers of pituitary venous dilution during bilateral simultaneous inferior petrosal sinus catheterization in Cushing's syndrome: Is combined CRF and TRH stimulation of value?. Clinical Endocrinology.

[cit19] Findling James W., Kehoe Michael E., Raff Hershel (2004). Identification of Patients with Cushing’s Disease with Negative Pituitary Adrenocorticotropin Gradients during Inferior Petrosal Sinus Sampling: Prolactin as an Index of Pituitary Venous Effluent. The Journal of Clinical Endocrinology & Metabolism.

[cit20] Mulligan Guy B., Eray Esin, Faiman Charles, Gupta Manjula, Pineyro Maria M., Makdissi Antoine, Suh John H., Masaryk Thomas J., Prayson Richard, Weil Robert J., Hamrahian Amir H. (2010). Reduction of False-Negative Results in Inferior Petrosal Sinus Sampling with Simultaneous Prolactin and Corticotropin Measurement. Endocrine Practice.

[cit21] Heaney Anthony P., Melmed Shlomo (2004). Molecular targets in pituitary tumours. Nature Reviews Cancer.

[cit22] Sharma S. T., Raff H., Nieman L. K. (2011). Prolactin as a Marker of Successful Catheterization during IPSS in Patients with ACTH-Dependent Cushing's Syndrome. The Journal of Clinical Endocrinology & Metabolism.

[cit23] Belaia Zh E, Rozhinskaia L Ia, Mel'nichenko G A, Sitkin I I, Dzeranova L K, Marova E I, Vaks V V, Vorontsov A V, Il'in A V, Kolesnikova G S, Dedov I I (2014). The role of prolactin gradient and normalized ACTH/prolactin ratio in the improvement of sensitivity and specificity of selective blood sampling from inferior petrosal sinuses for differential diagnostics of ACTH-dependent hypercorticism. Problems of Endocrinology.

[cit24] Sitkin Ivan I., Malygina Anastasia A., Belaya Zhanna E., Rozhinskaya Lyudmila Y., Buryakina Svetlana A. (2018). Inferior petrosal sinus sampling in differential diagnosis of ACTH-dependent hypercortisolism. Endocrine Surgery.

[cit25] Strebkova N.A., Udalova N.V. Bolezn' Itsenko-Kushinga u detei i podrostkov // Farmateka. — 2009. — №3. — S. 48-52.

[cit26] Chen Shi, Chen Kang, Lu Lin, Zhang Xiaobo, Tong Anli, Pan Hui, Zhu Huijuan, Lu Zhaolin (2018). The effects of sampling lateralization on bilateral inferior petrosal sinus sampling and desmopressin stimulation test for pediatric Cushing's disease. Endocrine.

